# Randomly auditing research labs could be an affordable way to improve research quality: A simulation study

**DOI:** 10.1371/journal.pone.0195613

**Published:** 2018-04-12

**Authors:** Adrian G. Barnett, Pauline Zardo, Nicholas Graves

**Affiliations:** 1 School of Public Health and Social Work, Queensland University of Technology, Brisbane, Australia; 2 Data & Policy, Faculty of Law & Digital Media Research Centre, Queensland University of Technology, Gardens Point, QLD, Australia; Tilburg University, NETHERLANDS

## Abstract

The “publish or perish” incentive drives many researchers to increase the quantity of their papers at the cost of quality. Lowering quality increases the number of false positive errors which is a key cause of the reproducibility crisis. We adapted a previously published simulation of the research world where labs that produce many papers are more likely to have “child” labs that inherit their characteristics. This selection creates a competitive spiral that favours quantity over quality. To try to halt the competitive spiral we added random audits that could detect and remove labs with a high proportion of false positives, and also improved the behaviour of “child” and “parent” labs who increased their effort and so lowered their probability of making a false positive error. Without auditing, only 0.2% of simulations did not experience the competitive spiral, defined by a convergence to the highest possible false positive probability. Auditing 1.35% of papers avoided the competitive spiral in 71% of simulations, and auditing 1.94% of papers in 95% of simulations. Audits worked best when they were only applied to established labs with 50 or more papers compared with labs with 25 or more papers. Adding a ±20% random error to the number of false positives to simulate peer reviewer error did not reduce the audits’ efficacy. The main benefit of the audits was via the increase in effort in “child” and “parent” labs. Audits improved the literature by reducing the number of false positives from 30.2 per 100 papers to 12.3 per 100 papers. Auditing 1.94% of papers would cost an estimated $15.9 million per year if applied to papers produced by National Institutes of Health funding. Our simulation greatly simplifies the research world and there are many unanswered questions about if and how audits would work that can only be addressed by a trial of an audit.

## Introduction

The estimated global annual health and medical research budget (including public and private funding) in 2012 was USD $268 billion [1]. Using published evidence Chalmers and Glasziou estimated that 85% of research studies give no return on that investment, meaning $228 billion per year is currently wasted [2], where waste is defined as research that produces no results or flawed results. The three causes of waste in their calculations were failure to publish, failure to adequately report the research, and failure to avoid critical design flaws.

Waste could be greatly reduced if researchers adhered to established standards of good research practice and avoided sloppy practices such as p-hacking to find statistically significant results with inflated effect sizes [3]. High paper numbers and journal impact factors are crucial to a researcher’s career progression [4, 5], which drives some researchers to sacrifice quality for quantity. The problem has long been recognised and Altman made the case in 1994 for researchers to focus on quality rather than quantity when he said, “We need less research, better research, and research done for the right reasons” [6]. Reducing waste in research would have tremendous value for society, as well-conducted health and medical research can transform lives and realise large health and economic benefits.

The current reward system based on quantity of output is deeply ingrained and it will need a large change to shift the focus to quality. To date, citations have often been used as a proxy for quality in assessments of research excellence. A key issue is that research quantity (e.g., the total number of published papers) and existing citation-based quality metrics, such as the impact factor and h-index, are easy to measure. Such measures are therefore often used for key decisions such as funding, tenure and promotion when large numbers of competing researchers need to be quickly compared [5, 7]. But these metrics can be gamed [8] and as the designers of a recent quality measure pointed out [9], “[no] metric should be taken as a substitute for the actual reading of a paper in determining its quality.” However, a detailed reading of the quality of a researcher’s work is time-consuming [10] and funding agencies already struggle to find reviewers because of the time commitment needed to do a good job [11].

We investigate whether using random audits of research groups can provide an accurate and affordable measure of quality. The idea is that audits might improve overall research practice because of the concern of being randomly selected. Random audits are used effectively in other systems where complete checking is impossible such as income tax and drunk driving [12, 13]. Auditing—if well designed—could also provide an incentive for quality over quantity of papers. Auditors would value a smaller number of papers that actually change practice or improve people’s lives, over a large number of poorly done papers. The economic signal is that quality is valued more than quantity.

Random audits have already been applied and discussed in research. The *Science Foundation Ireland* recently introduced random audits of laboratory scientists to check on experimental details and data [14]. A 2015 article in *Nature* supported random audits to verify good research practice [15]. Random audits of submitted papers by groups of journal editors was suggested by Rennie in 1988 [16], and Edward Huth, the then editor of the *Annals of Internal Medicine* said, “I think scientists would willingly accept random audit of their work by the government in exchange for the privilege of being eligible for federal grants. It would be better for everyone concerned.”

This paper does not design the audits in detail, but we anticipate that the sampling frame for random selection would be all research groups who recently received public funding, e.g., in the previous five years. We do not see audits as a means of catching fraudulent researchers [17], instead we see them as being used to provide a strong incentive to comply with good research practice and encourage behaviour that avoids the common causes of research waste [2]. Audits would focus on how researchers achieved their results, and be less concerned with metrics such as the number of papers in journals with high impact factors. An audit could take a month or more and involve multiple auditors who themselves would be experienced researchers with no conflicts of interest and who would be paid for their time. Auditors would be able to ask researchers for further information, such as the status of a funded project which has not been published. Audits would not involve re-running experiments to test if a published result was reproducible as this would take too much time [18]. Auditors would be able to examine measures of quality that metrics cannot, such as originality, reproducibility and translation into practice. Auditors would be able to compile information that indicated a systemic problem with a lab, such as the percent of inaccurate images [19] and the percent of retracted papers. The variety and depth of data that an audit could examine would make them difficult to game.

The aim of this paper is to provide preliminary evidence for the feasibility of a system of random audits by estimating how many papers would need to be audited to improve the overall quality of research. We also examine how much audits might cost.

## Materials and methods

We adapted the methods of a study by Smaldino and McElreath (2016) that examined research quality over time by simulating competing laboratories [10]. The aim of that study was to show how bad research practices can spread when selection favours laboratories that pursue quantity over quality. The methods used are well suited to our purposes because: i) laboratories have characteristics that control their research output, and ii) the overall quality of papers is measured using the false discovery rate. A false discovery is made when a laboratory declares a hypothesis to be true which we know to be false. Such mistakes increase when laboratories reduce their effort, and reducing effort allows a laboratory to test more hypotheses and so increase their output. Laboratories with the greatest output are more likely to have “child” laboratories who inherit their level of effort. Hence there is a natural selection for increased output and reduced effort which increases the false discovery rate.

We first successfully replicated the results of the original paper, which found a decline in research quality over time due to a selection for quantity over quality [10]. Then we simulated the impact of random audits to see if the decline in quality could be avoided. All the R code to run the simulations is available on github (https://github.com/agbarnett/taxinspect). We used R version 3.3.1 [20].

### Simulations

We simulated a research world of 100 labs. Simulated labs (indexed by *i* = 1, …, 100) choose to tackle a new hypothesis with probability: 1 − *η*log_10_ *e*_*i*_, where *e*_*i*_ which ranges from 1 to 100 is the lab’s effort which dictates the amount of time it spends on a hypothesis. We used *η* = 0.2, which meant labs that exerted less effort were more likely to take on a new hypothesis which is consistent with them having more time. An effort of *e*_*i*_ = 100 gives a probability of 0.6 of taking on a new hypothesis and an effort of *e*_*i*_ = 20 gives a probability of 0.8.

Simulated labs are randomly assigned a hypothesis to test which is true with probability 0.1 [21], so most hypotheses are false which represents the complexities of human chemistry, biology and behaviour. We do not know what the true probability is and this estimate of 0.1 is speculative. If we only considered research which had already progressed to phase III clinical trials, then the probability would be closer to 0.5 [22].

A false positive error is when a false hypothesis is incorrectly perceived to be correct following analysis. A simulated lab makes a false positive error with probability:

$$\begin{array}{c} \alpha_{i}=\frac{W_{i}}{1+\left(1-W_{i}\right)e_{i}} \end{array}$$ (1)

where *W*_*i*_ (range from 0 to 1) is the power of a lab to correctly detect a true hypothesis and reflects the methods used in the lab. We fixed *W* to 0.8 for all labs as we were more interested in changes in effort. Labs receive a pay-off for every positive novel result they publish, regardless of its accuracy.

To start the simulation we gave all 100 labs an initial effort of *e*_0_ = 75 out of 100, which gives a false positive error of *α* = 0.05. So all labs begin with the commonly used assumptions of sample size calculations of a power of 0.80 and type I error of 0.05 [23].

Once all the labs have selected and tested their hypotheses, a lab is randomly removed and is replaced by a “child” of a surviving lab. Ten of the 100 labs are randomly selected and then the oldest lab is removed. Ten of the surviving 99 labs are randomly selected and the lab with the highest cumulative pay-off is selected to have one child. The child inherits the parent lab’s effort (*e*_*i*_), but with probability 0.01 the inheritance occurs with mutation, so the child inherits slightly altered characteristics, $e_{i}^{\text{child}}=e_{i}^{\text{parent}}+\psi$, where *ψ* is randomly selected from a Normal distribution with mean zero and standard deviation 1. The birth-and-death process mimics the real world by both gradually removing older labs due to retirement, and rewarding high achieving labs with an element of randomness [24]. A “research cycle” is the complete process of hypotheses selection, testing, pay-off, birth and death for all 100 labs.

### Audits of false positives

The simulation formulae and assumptions presented thus far closely follow those used in Smaldino and McElreath (2016). What we present below is a departure from their model.

After the first 100 research cycles, audits are run every *j*th research cycle and we varied *j* in order to estimate how many audits were needed. At every audit one lab was randomly selected from those labs that had not previously been audited and had produced *n* = 50 papers or more. This restriction was used to avoid auditing labs with little available data.

We assumed that the auditors could establish a perfect view of a lab’s publication history and so could calculate their true false positive probability. This is a key assumption and we consider it in detail in the discussion. We also relaxed this assumption in a sensitivity analysis (see below). We assumed that auditors had knowledge of the overall false positive probability of all labs, and a lab was removed if their false positive probability was in the upper third of this distribution, so if *α*_*i*_ > *c* where *c* is chosen from the empirical cumulative distribution function *F*(*c*) = 0.67, so *c* varies over time. This means one in every three audits ends with a removal, which we thought was strong enough to send a clear signal to other labs, but also that a lab with a median false positive probability was not removed. The minimum value for *c* was 0.05 to avoid removing labs that had a low false positive probability. If a lab was removed then a replacement was made using same procedure described above for generating child labs.

Audited labs increase their effort by *el*^+^ > 0, and there was also an effect on the lab’s parents and children who increased their effort by *en*^+^. This assumes that labs in the same “family” remain networked and that the impact of an audit would stretch beyond the selected lab. Our reasoning was that such labs usually remain in contact, possibly working on joint projects and exchanging staff. We began with *el*^+^ = *en*^+^ = 5, which for a starting effort of *e* = 75 reduces the probability of taking on a new hypothesis by 0.006 (0.625 probability for *e* = 75 versus 0.619 for *e* = 80). An increase in effort of 5 is relatively large, but many of the changes that researchers need to make are relatively simple improvements such as adhering to established methods to avoid “common pitfalls” [25]. We also know of an example where an audit of pharmaceutical companies lead to an improvement in research behaviour [26].

### Audits of mistakes

The above simulation relies on the auditors being able to identify false positives. However, this is likely to be difficult because negative studies that overturn positive findings are often not published [27], hence there would be no indication that a positive finding was incorrect. Also, for recent papers there would not have been time for a replication study to be published.

As an alternative we simulated auditors looking for mistakes in published papers. Three examples of mistakes or poor research practice that auditors could look for are:

- “Outcome switching” where the outcomes in the published results of a trial are different to those stated in the protocol [28]. This switching is generally used to give a result that is “statistically significant” and hence more likely to be accepted for publication. Outcome switching exaggerates effects and greatly increases the chance of a false positive finding. The COMPARE project has checked 67 recent trials in the top five medical journals and found that 58 (86%) reported outcomes that were different to those stated in the protocol [29].
- Common mistakes in study design. A review of 1,286 trials found 556 (43%) had at least one study design element with a high risk of bias in either: sequence generation, allocation concealment, blinding of participants and personnel, blinding of outcome assessment and incomplete outcome data [30].
- Contaminated cell lines in laboratory studies which can create meaningless results. A search of papers that cited a contaminated cell line found an estimated 0.7% of papers in the cell literature used a contaminated cell line [31].

We have not conducted a systematic review of what mistakes have been looked for in published papers, instead the above examples are meant to show that researchers can audit papers and subjectively count mistakes.

If a lab published a paper with a false positive error then we randomly simulated a mistake in that paper with probability 0.25. The three papers mentioned above have mistake probabilities between 0.007 and 0.86, although this was in all papers not conditional on making a false positive error. This demonstrates the great variety in the underlying probability of a mistake dependent on how it is classified. We chose 0.25 to reflect relatively serious errors that were neither very rare nor common.

A lab was removed if their cumulative proportion of mistakes (using their total number of papers as a denominator) was in the upper third of the empirical distribution of cumulative mistake proportions from all 100 labs. This was the same process as the audit based on the empirical distribution of false positives.

For this simulation we also estimated the potential benefits of auditing by counting the total number of false positives and papers with mistakes, and comparing these totals to a world without auditing. These results were scaled to per 100 papers.

### Audit costs

We estimated the audit costs by assuming that one auditor was needed for every 10 papers of the selected lab, and each auditor would be employed for one month. Auditors had an annual salary based on half being professors (USD $106K in 2011 using US Census data) and half being associate professors (USD $76K in 2011) giving an inflation-adjusted salary in 2016 of USD $105K [32]. To show the costs on a meaningful scale we calculated the costs per paper published (including audited and non-audited papers). To illustrate the scale of the audits we calculated the percentage of all published papers that were audited. We calculated averages for these statistics across all simulations together with non-parametric 95% confidence intervals using observed cumulative distributions from the simulations.

### Sensitivity analyses

We used a range of sensitivity analyses to examine which settings were most important and what assumptions were critical (Table 1). We first selected a baseline combination of *j*, *c* and *e*^+^ that was reasonably effective in terms of reducing false positives, and then examined the following changes:

- An increased cut-off to remove an audited lab from *c* = 0.67 to 0.95 to investigate a system that only removed the very worst performers.
- A cut-off of *c* = 1 so that no labs are removed to investigate the impact of removing labs.
- An increased post-audit effort from *el*^+^ = *en*^+^ = 5 to *el*^+^ = *en*^+^ = 10 to investigate a greater impact of auditing.
- A decreased post-audit effort from *el*^+^ = *en*^+^ = 5 to *el*^+^ = *en*^+^ = 0 to investigate no improvement in effort after auditing.
- An decreased post-audit effort from *el*^+^ = *en*^+^ = 5 to *el*^+^ = *en*^+^ = 1 to investigate an impact of auditing on a similar scale to the mutation change: *ψ* ∼ N(0, 1).
- No increase in post-audit effort for networked labs *en*^+^ = 0 compared with audited labs *el*^+^ = 5, and conversely no increase in post-audit effort for audited labs *el*^+^ = 0 compared with networked labs *en*^+^ = 5 to investigate the relative importance of the two increases in effort.
- Halving the minimum number of papers to be eligible for auditing from *n* = 50 to *n* = 25, to investigate whether auditing younger labs gave better results.
- Using initial efforts of *e*_0_ = 10 or 50 instead of 75 for all labs to investigate whether audits could be effective in a world with already high false positive probabilities.
- Adding error to auditors’ decisions to investigate an imperfect peer review process. We added error by randomly changing the number of observed false positives by ± 20%, so the error could either under- or over-estimate a lab’s quality. The size of the error was randomly generated using a Normal distribution with zero mean and standard deviation of (0.2∑_*i*_ *FP*_*i*_)/1.96, where *FP*_*i*_ is 1 if a published study *i* is a false positive and 0 otherwise.

**Table 1:** Parameters used in the simulations. Values in italics are the baseline scenario.

Parameter	Definition	Values tested
Parameters from Smaldino and McElreath (2016):		
*η*	Strength of the association between effort (*e*) and the probability of tackling a new hypothesis	*0.2*
*W*_*i*_	Power of a lab to correctly detect a true hypothesis	*0.8*
*e*_0_	Starting effort for labs	10, 50, *75*
*ψ*	Random mutation in effort from a standard Normal distribution	*N(0,1)*
Additional parameters for simulations presented here:		
*j*	Cycles per audit	50, *75*, 150, 200
*n*	Number of papers needed to be eligible for auditing	25, *50*
*el*^+^	Increased effort of audited labs	0, 1, *5*, 10
*en*^+^	Increased effort of networked labs	0, 0.5, 1, *5*, 10
*c*	Cut-off in cumulative distribution function at which labs are removed	*0.67*, 0.95, 1
Review error	Percent by which the number of false positives are randomly changed in order to simulate reviewer error	*0*, 20
Mistake probability	Mistake probability for papers with a false positive finding	*0.25*

Simulations ran for 800,000 research cycles and we recorded data every 8,000th cycle to give 100 records per simulation. We ran 500 simulations per scenario (combination of cycles per audit, *j*; cut-off, *c*; and increased effort, *el*^+^, *en*^+^). To show changes over time we plotted the average variable (e.g., false positive probability) of the 500 simulations and also plotted 20 randomly selected simulations to highlight the variation between simulations.

## Results

### Audits of false positives

**Fig 1:**
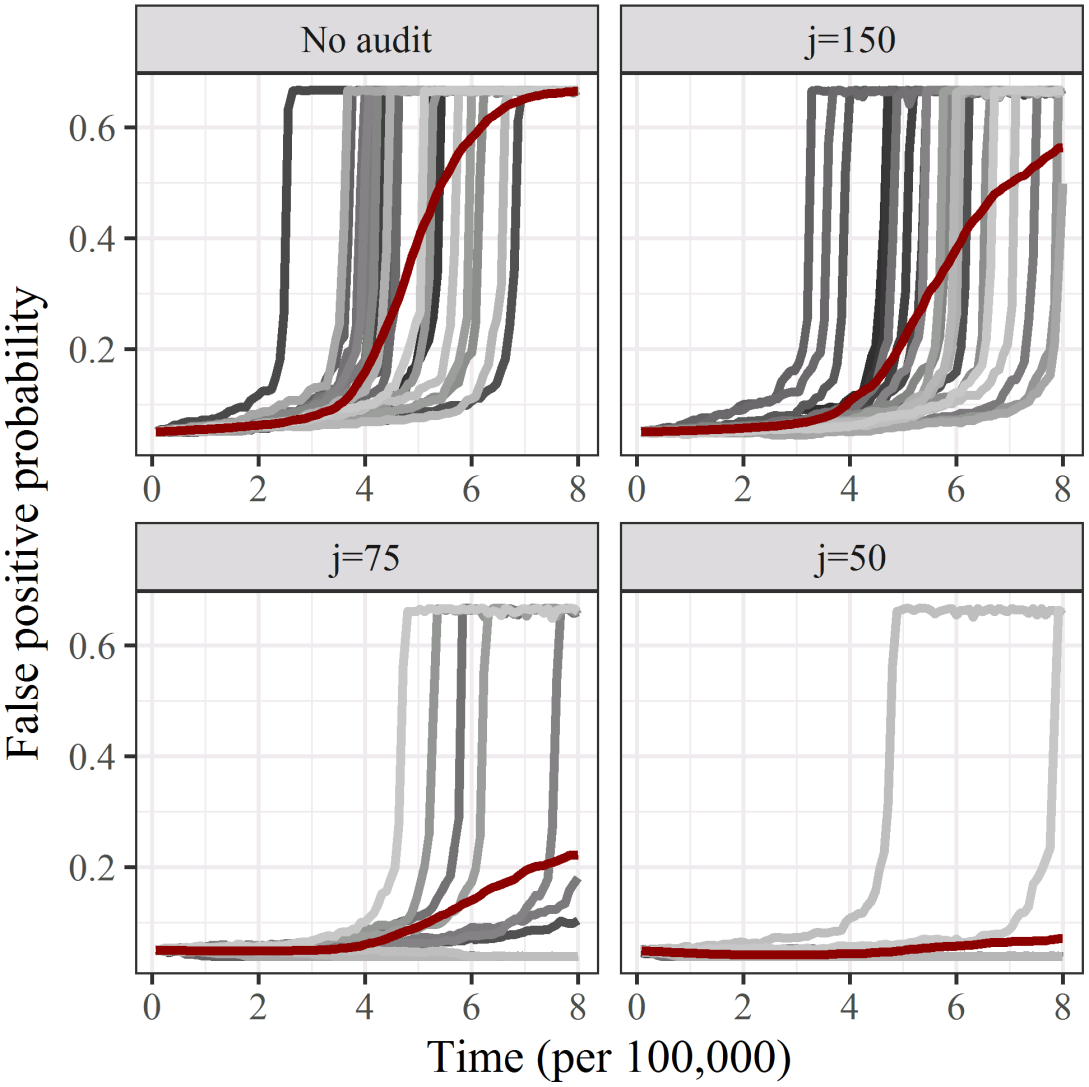
False positive probabilities by time (research cycle) for four levels of cycles per audit (*j*). The grey lines are 20 randomly selected simulations and the dark red line is the average of 500 simulations.

The false positive probabilities over time for four levels of auditing are plotted in Fig 1. Many simulations show a steady rise from the starting false positive probability of 0.05, followed by a very steep rise to the maximum false positive of 0.67 caused by labs reducing their effort to the minimum of 1 (this maximum probability comes from putting *e*_*i*_ = 1 into Eq (1)). The steep rise is caused by a strong selection for increased quantity when labs reduce their effort creating a competitive spiral. The average of the individual simulations shows a slower decline in quality over time because the competitive spiral starts at different times.

**Fig 2:**
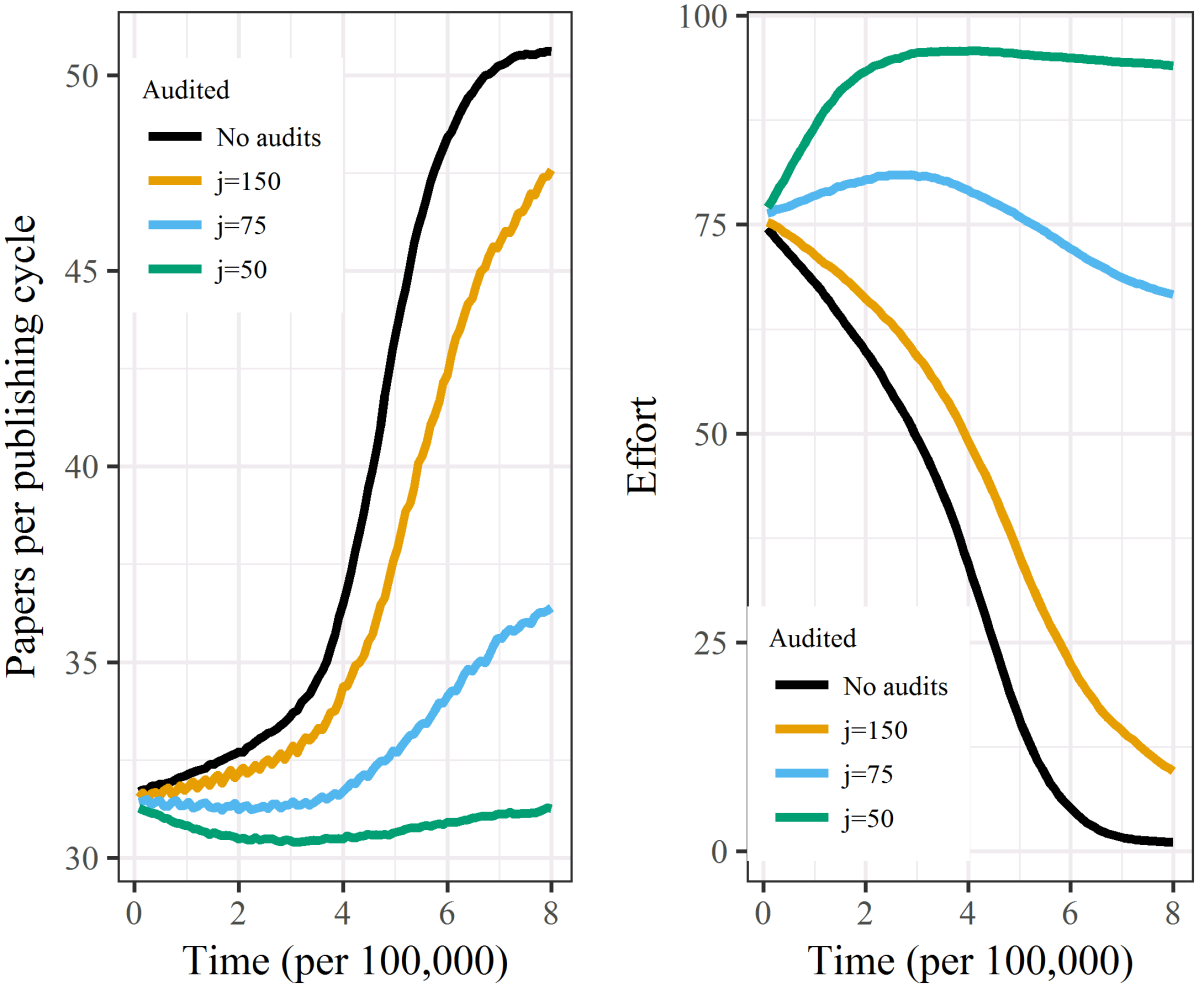
Mean number of papers produced (left) and mean effort (right) over time (research cycle) for 500 simulated labs for four levels of cycles per audit (*j*).

Without auditing only 0.2% of simulations did not experience the steep increase in false positives. Auditing 1.35% (*j* = 75) of papers avoided the steep increase in false positives in 71.4% of simulations, and auditing 1.94% (*j* = 50) of papers in 95.0% of simulations. Audits also decreased the volume of papers, as on average labs maintained their effort and reduced the number of new investigations (Fig 2).

The percent of papers audited and auditing costs are in Table 2. Auditing 1.94% of papers cost an extra USD $169 per published paper (95% confidence interval $152 to $171). This can be thought of as the surcharge added to every paper in order to pay for the audits and thus maintain a good overall research quality.

**Table 2:** Means and 95% confidence intervals for the percent of papers audited and costs per paper.

Cycles per audit, *j*	Percent of all papers audited	Cost per paper ($ USD)
50	1.94 (1.73, 1.96)	169 (152, 171)
75	1.35 (1.20, 1.40)	118 (105, 122)
150	0.59 (0.55, 0.64)	52 (48, 56)

The cost of auditing 1.94% of papers can be scaled up to national systems. In Australia, research funded by the National Health and Medical Research Council produced an estimated 4,138 papers per year between 2005 and 2009 [33], giving an annual auditing cost of USD $700,000 to audit 80 papers. Work funded by the US National Institutes of Health produced an estimated 94,000 papers in 2013 [34], giving an annual auditing cost of USD $15.9 million to audit 1,824 papers.

### Sensitivity analyses

**Fig 3:**
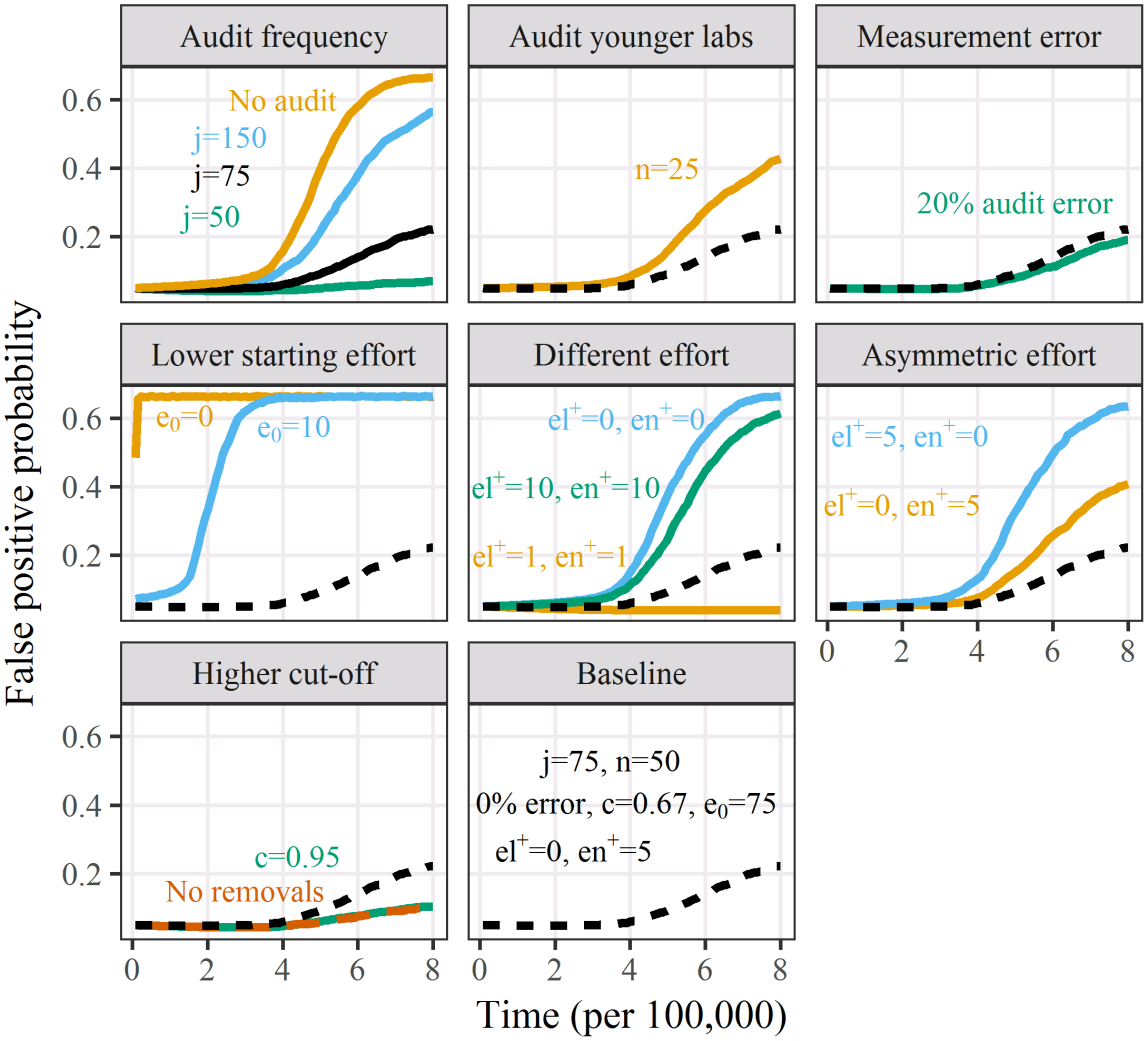
Mean false positive probabilities for 100 labs over time (research cycle) for the sensitivity analyses. *j* = 75 cycle per audit (Auditing 1.35% of all papers) is the baseline scenario with: starting effort *e*_0_ = 75; increased effort after auditing *el*^+^ = *en*^+^ = 5; percentile cut-off for lab removal *c* = 0.67; minimum papers *n* = 50; review error = 0%. All other results change one of these six parameters. The baseline scenario is shown as a dashed line in all the panels bar the top-left panel where it is a solid line.

The false positive probabilities by time (research cycle) for the sensitivity analyses are in Fig 3 and the percent of simulations that did not experience the competitive spiral are in Table 3. Audits did not work when the starting effort was low across all labs (*e*_0_ = 10 or 50). Having no impact on effort using *el*^+^ = *en*^+^ = 0 gave almost the same results as no auditing. The increase in effort can be too great, as using *el*^+^ = *en*^+^ = 10 led to far worse results than *el*^+^ = *en*^+^ = 5. This was because the decline in paper numbers by audited labs and their network using an increased effort of 10 made them less competitive on quantity and this loss was not compensated for by their improved false positive probability. This illustrates the tension between quantity and quality. The best results were for the smallest increase in effort *el*^+^ = *en*^+^ = 1 and this change in effort was on a similar scale to the mutation rate from the standard Normal distribution for *ψ*.

**Table 3:** Percent of simulations that did not experience the competitive spiral. Results from 16 scenarios. Results ordered by best (highest percent) to worst performing scenarios. See Table 1 for the key to the parameters.

*j*	*e*_0_	*en*^+^	*el*^+^	*n*	*c*	Review error (%)	Audits based on	Percent
75	75	1	1	50	0.67	0	False positives	100.0
50	75	5	5	50	0.67	0	False positives	95.0
75	75	5	5	50	1.00	0	False positives	90.0
75	75	5	5	50	0.95	0	False positives	89.6
75	75	5	5	50	0.67	20	False positives	76.4
75	75	5	5	50	0.67	0	False positives	71.4
200	75	0.5	1	50	0.67	0	Mistakes	52.8
75	75	5	0	50	0.67	0	False positives	42.6
75	75	5	5	25	0.67	0	False positives	39.5
150	75	5	5	50	0.67	0	False positives	18.0
75	75	10	10	50	0.67	0	False positives	9.3
75	75	0	5	50	0.67	0	False positives	5.3
75	75	0	0	50	0.67	0	False positives	0.4
No audits	75	–	–	–	–	–	–	0.2
75	10	5	5	50	0.67	0	False positives	0.0
75	50	5	5	50	0.67	0	False positives	0.0

The results for the asymmetric increase in effort between audited labs and their network (*el*^+^ ≠ *en*^+^) indicate that the increase in effort for audited and networked labs were both important, but the increase for audited labs had the more beneficial effect.

Audits worked better when only 1 in 20 labs (*c* = 0.95) or even no labs were removed, compared with removing 1 in 3 labs. This indicates that removing labs may not be necessary and it is the increasing effort of the audited labs and their network that maintains quality.

Adding a 20% measurement error had no impact on the results, showing that the audits were relatively robust to peer review error. This is because we used a relatively large sample size as the minimum paper numbers was *n* = 50. Including younger labs in the audit (*n* = 25) produced worse results because this reduced the sample size and meant that labs with a relatively small number of false positives could be removed, which meant that some good labs were removed by chance.

### Audits of mistakes

Based on the previous simulations we used an increased effort of *el*^+^ = 1 for audited labs and *en*^+^ = 0.5 for networked labs to reduce the influence of the network assumption. We used an increased number of cycles per audit of *j* = 200 compared with *j* = 50 to 150 in the audits of false positives because the effort of *el*^+^ = 1 and *en*^+^ = 0.5 meant individual audits were more effective than an increased effort of 5.

**Fig 4:**
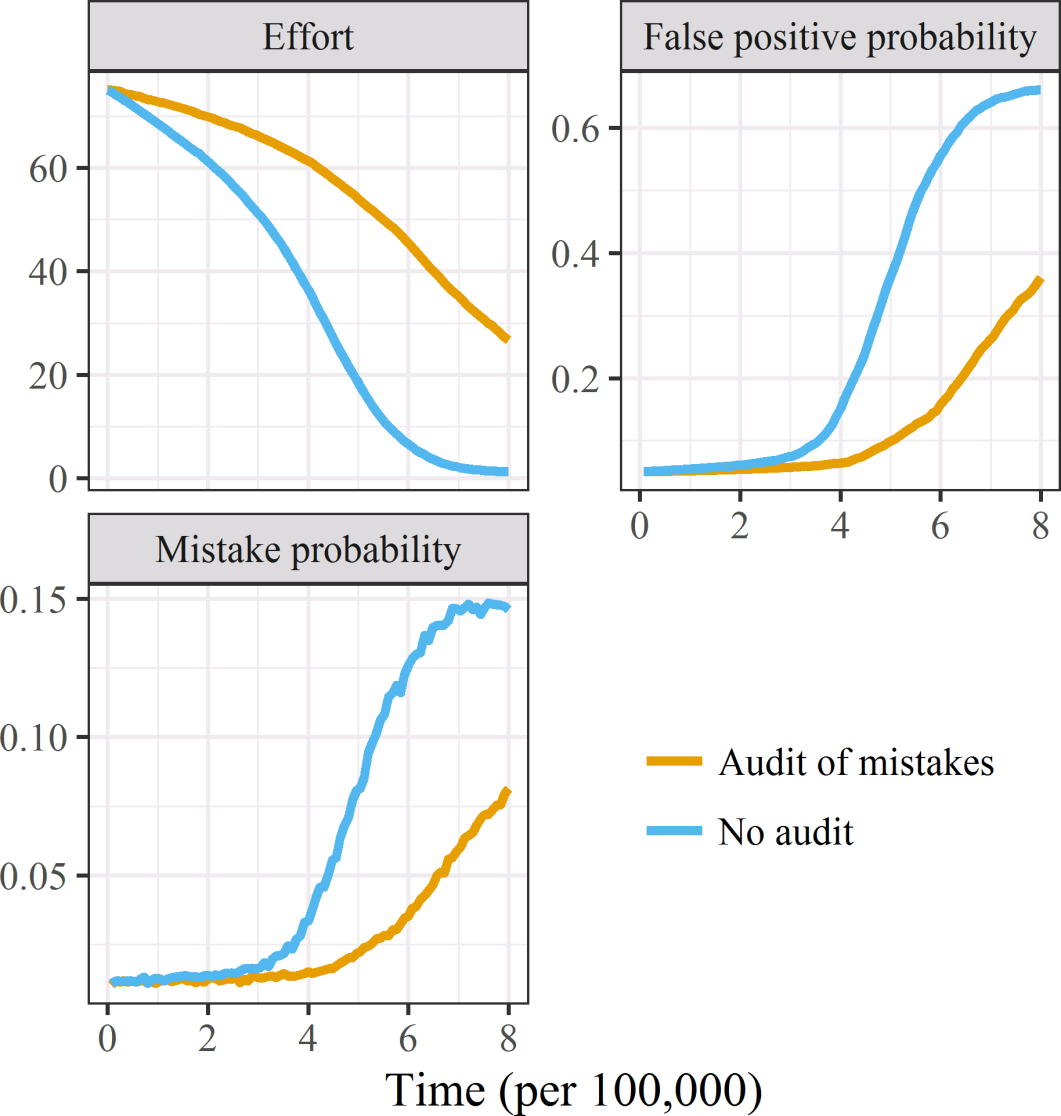
Means of effort, false positive and mistake probabilities for 100 labs over time (research cycle) with and without audits based on finding mistakes in published papers.

The results over time comparing audits of counting mistakes to no audits are in Fig 4. Using audits of mistakes slowed the decline in effort and slowed the increase in published false positives and mistakes.

We show the estimated benefits of using audits on the literature in Table 4. Without audits there was an average of 30.2 false positives per 100 papers, but with audits this decreased to 12.3 false positives per 100 papers. The numbers of mistakes reduced from an average of 8.1 per 100 papers without auditing to 3.8 with auditing.

**Table 4:** Means and 95% confidence intervals for the number of false positives and number of mistakes per 100 papers. Results for two simulations, one without audits and one with audits with *j* = 200, *el*^+^ = 1 and *en*^+^ = 0.5.

	False positives	Mistakes
No audit	30.2 (12.3 to 41.5)	8.1 (3.8 to 10.7)
Audits	12.3 (4.2 to 34.9)	3.8 (1.7 to 9.2)

## Discussion

In all but 0.2% of the simulations with no auditing there was a large decline in research quality as measured by the false discovery probability (Fig 1). This occurred because quantity was rewarded with no feedback on quality, a result already shown by two previous simulations of the research world [10, 35]. Smaldino and McElreath (2016) found that the decline could not be reversed by randomly reproducing some papers and severely punishing those labs that made a positive finding that failed to replicate [10]. This approach did not work because removing individual poor performers was not sufficient, as there were too many other poor performers who continued to be rewarded. Hence our approach of using audits aimed to have a wider impact by including labs that were connected to the audited lab because they were a “child” or “parent” of the audited lab. We assumed that these connected labs would react to the audit because of the concern that they could be audited next. Our sensitivity analyses (Fig 3) showed the value of the network effect as the audits did not work as well when the increased effort for the networked labs was set to zero (*en*^+^ = 0).

Another recent simulation study examined: i) using funding to reward diligent labs that kept their false publication rate low, and ii) not funding fraudulent labs that knowingly published false positive results in order to increase their publication output [36]. A combination of a high fraud detection rate (probability over 0.75) and high reward for diligent labs was relatively successful in maintaining the proportion of reproducible results, especially compared with scenarios where all labs were rewarded based simply on their positive output.

Interestingly our audits worked better when no labs were removed, meaning this punitive step may not be needed. Removing labs would be controversial in practice as it would require actions such as refusing further funding. However, in reality labs may only increase their effort if the audits had a potentially serious consequence. Reputation is central to academic culture and career progression [37]. It is therefore possible that the threat of receiving a poor report by the auditors may be enough to improve behaviour given the potential damage that a poor report could do to the lab’s chances of winning funding. The related simulation mentioned in the previous paragraph assumed that a lab where fraud was detected in published papers could be denied funding [36]. In practice it is also likely that larger labs are run by more senior academics, for whom the reputational risk of a poor audit report would be significant.

An interesting result was that smaller improvements in practice post-audit (*el*^+^ = *en*^+^ = 1) gave much better overall results than larger improvements (*el*^+^ = *en*^+^ = 10). This is because when labs made a large improvement in their effort, they also greatly decreased their output and this decrease made them less competitive. This may mirror real life where labs that make dramatic shifts in practice from the norm can be considered as too unorthodox. A recent systematic review found “suggestive” evidence that peer reviewers are anti-innovative when deciding on funding [38], and the most successful strategy for winning funding may be sticking close to the status quo.

Our results showed the importance of examining the entire work of a lab rather than a single paper in a reproducibility exercise as simulated by Smaldino and McElreath [10]. A related simulation found a similar result, as they found that making more published studies count for researchers’ careers increased the total scientific value of research [35]. A systematic review of using peer review to judge individual papers found that, “little empirical evidence is available to support the use of editorial peer review as a mechanism to ensure quality of biomedical research” [39]. However, audits should be more reliable because they examine multiple papers rather than a single paper, and we only audited labs with a minimum sample size of *n* = 50 papers. In the sensitivity analysis (Fig 3) halving the sample size of papers to *n* = 25 greatly reduced the efficacy of the audits, whilst adding an auditing error of 20% did not reduce efficacy. These results indicate that audits would work best when applied to older labs with a large sample size of published papers on which to judge the lab’s overall quality. Also larger labs have, on average, more child labs and hence the impact of the audit is greater.

Audits may have political appeal because they have had some success in other policy areas and have a strong face validity that can be explained to the public. A 2011 UK House of Commons scientific and technology committee review of peer review, “found the general oversight of research integrity in the UK to be unsatisfactory” [40]. There may be support for change because the issue of research waste has received widespread attention and is being discussed by key international stakeholders [41] and a survey of over 1,500 researchers found that most agreed that there was a reproducibility crisis [42]. An auditing system would likely face strong resistance from some researchers and institutions, and may be seen as taking money away from actual research and being another layer of bureaucracy in the “hyper-regulation of research” [43]. However, in our simulations audits secured the value of the larger investment in research as they greatly reduced false positive errors (Table 4), improving the evidence-base. Reliability of research findings has been identified as one of the top five barriers to use of research health policy decision-making in systematic reviews of evidence on translation of research to policy [44]. Therefore, improving the reliability of research findings can in turn lead to better health policy decisions and subsequent health and economic benefits.

Automated systems are being used to evaluate published papers and rank laboratories [45]. Automated systems have the great advantages of being low cost, instantly updatable and being deployable as a census rather than a random sample. However, there are important outcomes that automated systems cannot detect and need to be examined by humans. Automated systems could be used to flag potential problem laboratories for subsequent human auditing, and this two-stage approach would likely be cheaper than a human-only system.

### Limitations

Our simulations greatly simplify the research world. There are 26 key parameters that we can set (see R code https://github.com/agbarnett/taxinspect) and we did not investigate every combination, but instead chose a subset of combinations based on group discussion amongst the authors. Our assumptions may not translate into real life [46] and we have not fully developed how the audit would work in practice. An auditing system could first be trialled without releasing any results in order to test its ability and improve its design.

Using audits would be a huge change to the current system and although the costs are relatively small when viewed on a per paper scale (Table 2) the auditing system would need to be nationally supported by researchers, universities and funders. Audits would likely be very unpopular with some researchers and audited labs would likely experience stress and uncertainty during the process. If the audits were perceived to make mistakes or made genuine mistakes then their value could be undermined. Labs that were closed down by an audit may be politically well connected and run by powerful professors who would lobby for their reinstatement and publicly decry the accuracy of the audit. Members of a closed lab may move to other labs and continue their poor research practices there.

Our most questionable assumption is the ability of the auditors to perfectly establish a laboratory’s history of false positives. Peer review is imperfect and problems could occur with inadequate experience, undeclared conflicts of interest and allegiance bias [47, 48]. However, peer review of an entire publication history should have less error than reviewing individual papers by virtue of the larger sample size and ability to see a lab’s overall philosophy and direction. For example, a study of funding applications found a greater agreement between independent reviewers when judging fellowship applications—where the key task is judging past performance—compared with judging project applications—where the key task is judging new proposals [49]. We note that false positives are uncovered in research, as demonstrated by a review of 1,344 articles which found 146 papers where a current medical practice was reversed. [50]. Hence it is possible that an audit would find positive findings from authors that had been disproved by studies with a greater statistical power or better design.

The level of audits used did not work when effort was low and false positive probabilities were high, which may be the current situation [18, 51]. Hence the real world may need far more audits and so be far more costly than our estimates. The real costs would also be somewhat higher because we did not include the costs of establishing and governing the audit system.

There is a danger that audits would harm average performers by damning with faint praise. If a lab was given an average report then that may be perceived to be inadequate in a world where researchers must strive for “excellence” despite this word having little meaning in research [52]. This could be avoided by only making public the audit reports from labs that failed to meet the cut-off, but this would reduce transparency and likely increase mistrust in what would already be an unpopular system. It is important to consider limitations regarding academic perceptions of the value of a new performance assessment approach in light of existing systems of research performance assessment, which are also widely recognised by the academic community as imperfect [53].

We assumed that once a lab had been audited it would never be audited again in its lifetime. This was done on the basis that it would be perceived as being inequitable if the same lab was audited twice within a few years. However, being free of the threat of audits would create an incentive to decrease effort, or at least not improve. In reality a balance may need to be struck between equity and incentives, possibly by using a grace period after an audit.

Our simulation that examined mistakes assumed that mistakes only occurred in papers with a false positive error, whereas it would be more realistic to also allow mistakes in papers without a false positive error but with a lower probability.

### Further research

Ours is a naive model of the research world which could be further developed to be more sophisticated or test other reactions to the audits, and all our code is freely available (https://github.com/agbarnett/taxinspect). One interesting change is a non-linear increase in effort (*el*^+^ and/or *en*^+^) depending on the audited lab’s current effort. This could be used to model a smaller increase in effort for labs that already had a high effort, assuming their potential to improve practice was smaller. Alternatively a smaller change in effort for labs with a low current effort could be modelled, assuming that such labs were chronic under-performers who would not change practice.

Our simulations tell us little of what would happen in the real world, so the next logical step is to trial an audit to see what can be achieved and what problems occur. A small number of labs could be randomly selected using a national sampling frame of recent winners of funding. We think that even a very small sample of five to ten labs would be useful. This pilot would reveal the reaction of labs to being selected, the costs and difficulties of peer review, and whether the reviewers could make judgments on characteristics that cannot be captured by metrics, such as originality, reproducibility and translation into practice.

## Conclusion

The aim of this paper is not to advocate for the use of audits to solve the current issues in research. Instead our aim was to provide an initial investigation into whether audits could affordably maintain research quality in a competitive research world where quantity is highly prized. The simulations support the idea that a detailed scrutiny of a relatively small proportion of all research output could maintain overall research quality by allowing research groups that prioritise quality to survive. However, there are many practical and political issues with using audits that we have not addressed. Audits would be a major change to the current system, but such major changes may need to be considered given the many current problems with research quality.
